# Early detection of the impact of combined taxane and carboplatin treatment on autonomic nerves in patients with cervical cancer: Measurement of heart rate variability

**DOI:** 10.3389/fphys.2023.1126057

**Published:** 2023-02-28

**Authors:** Jian Liu, Weizheng Guan, Yilin Sun, Yuling Wang, Guangqiao Li, Sai Zhang, Bo Shi

**Affiliations:** ^1^ Department of Gynecologic Oncology, First Affiliated Hospital, Bengbu Medical College, Bengbu, Anhui, China; ^2^ School of Medical Imaging, Bengbu Medical College, Bengbu, Anhui, China; ^3^ Anhui Key Laboratory of Computational Medicine and Intelligent Health, Bengbu Medical College, Bengbu, Anhui, China

**Keywords:** heart rate variability, autonomic nervous system, cervical cancer, chemotherapy, taxane

## Abstract

**Background:** Previous studies have shown that heart rate variability (HRV) analysis is a sensitive indicator of chemotherapy-induced cardiotoxicity. However, most studies to date have observed long-term effects using long-term analyses. The main purpose of this study was to evaluate the acute effect of chemotherapy on the cardiac autonomic nervous system (ANS) in patients with cervical cancer (CC) by examining short-term HRV.

**Methods:** Fifty patients with CC admitted to the Department of Gynecology and Oncology of the First Affiliated Hospital of Bengbu Medical College were enrolled in the study. Based on their chemotherapy regimens, the patients were divided into a DC group (docetaxel + carboplatin) and a TC group (paclitaxel + carboplatin). A 5-min resting electrocardiogram (ECG) was collected before and the day after chemotherapy: the time domain (standard deviation of normal-to-normal intervals (SDNN) and root mean square of successive differences (RMSSD)) and frequency domain (low-frequency power (LF), high-frequency power (HF), and (LF/HF)) parameters were analyzed, and the differences before and after chemotherapy were compared.

**Results:** The results showed that SDNN, RMSSD and HF were significantly higher in the DC and TC groups after chemotherapy than before (*p* < 0.05, Cohen’s *d* > 0.5). In addition, LF was significantly higher after TC than before chemotherapy (*p* < 0.05, Cohen’s *d* > 0.3), and LF/HF was significantly lower after DC than before chemotherapy (*p* < 0.05, Cohen’s *d* > 0.5).

**Conclusion:** Chemotherapy combining taxane and carboplatin can increase the HRV of CC patients in the short term, and HRV may be a sensitive tool for the early detection of chemotherapy-induced cardiac ANS perturbations.

## Introduction

Cervical cancer (CC) is a common gynecological cancer (Sung et al., 2021). Chemotherapy, an important means of cancer treatment, chemotherapy can effectively reduce the tumor burden of cancer patients and prolong their survival time (Monsuez et al., 2010; Regalado Porras et al., 2018; Gabani et al., 2021). Nevertheless, the toxic side effects of chemotherapy cannot be ignored. Cardiotoxicity is one of the clinically recognized side effects of chemotherapy. Chemotherapy-induced cardiotoxicity can develop over time in an acute, subacute or chronic manner, with acute or subacute cardiotoxicity appearing at any time from the start of treatment to 2 weeks after the end of treatment (Madeddu et al., 2016). A large cohort study of 2,625 cancer patients who received chemotherapy showed that the overall incidence of cardiotoxicity was 9% during a median follow-up of 5.2 years (Cardinale et al., 2015). Thus, the early detection of cardiac function in patients with chemotherapy can help to prevent and identify the occurrence of cardiotoxicity and guide and improve the direction of subsequent treatment to maximize patient cardiac safety.

Electrocardiogram (ECG) is a routine method to identify cardiotoxicity. However, the abnormalities found on ECG may be interfered with by numerous factors, and the specificity of cardiotoxicity detection is low. Imaging methods, such as echocardiography, cardiac magnetic resonance (CMR), and multigated radionuclide angiography (MUGA), are also commonly used to evaluate cardiotoxicity in clinical practice (Tan and Scherrer-Crosbie, 2012; Reuvekamp et al., 2016; Jordan and Hundley, 2019; Tak et al., 2020; Siddiqui et al., 2022). However, the accuracy of ultrasound examination is affected by the physician’s technology and image quality (Jerusalem et al., 2019), there are many contraindications in magnetic resonance examination (Christian et al., 2012), and MUGA can cause radiation damage to patients (Bikiewicz et al., 2021). As a result, imaging methods still have limitations in clinical use. Some cardiac biomarkers, such as B-type natriuretic peptide and cardiac troponin I, have also been considered for the detection of early cardiac injury during chemotherapy, but there is no consensus on the optimal time for biomarker measurement (Pudil et al., 2020; Semeraro et al., 2021). Therefore, determining the best way to assess early cardiotoxicity is still in the exploratory stage.

Currently, the mechanism of cardiac dysfunction is thought to involve abnormalities in autonomic nervous system (ANS) function (Teng et al., 2021). Heart rate variability (HRV), as an objective index to evaluate cardiac ANS regulation, is convenient and noninvasive (Patural et al., 2022; Rajendra Acharya et al., 2006). Several studies have suggested that HRV may be a sensitive indicator for evaluating chemotherapy-induced cardiotoxicity. For example, Frye et al. found that carotid artery stiffness was significantly higher and cardiovascular baroreflex sensitivity (cBRS) along with time- and frequency-domain HRV indices were significantly lower in cancer patients receiving chemotherapy compared to healthy controls; furthermore, cBRS correlated significantly with the low-frequency power of HRV (r = 0.66, *p* < 0.001) (Frye et al., 2018). Caru et al. compared HRV in acute lymphoblastic leukemia patients with different cumulative doses of doxorubicin. The results showed that the patients in the high-risk group had significantly altered HRV time-domain, frequency-domain and nonlinear indicators compared to the patients in the standard-risk group, suggesting that HRV is a sensitive indicator for detecting changes in cardiac ANS in patients (Caru et al., 2019).

At present, the use of HRV to detect chemotherapy-induced cardiotoxicity has been involved in a variety of cancers. However, few scholars have conducted research in this direction for CC patients. Therefore, this study used the traditional time- and frequency-domain indices of HRV to assess whether HRV can detect perturbations of the cardiac ANS in CC patients with chemotherapy and to provide new ideas for the evaluation of cardiotoxicity in CC patients receiving chemotherapy.

## Methods

### Subjects

The study subjects were 52 CC patients who received taxane combined with carboplatin adjuvant chemotherapy admitted to the Department of Gynecology and Oncology, the First Affiliated Hospital of Bengbu Medical College (Anhui, China), from December 2021 to October 2022.

The inclusion criteria were as follows: (1) patients with CC (pathological type of squamous and adenocarcinoma) confirmed by pathological examination; (2) patients who received chemotherapy and the chemotherapy regimen was taxane (docetaxel/paclitaxel) combined with carboplatin.

The exclusion criteria were as follows: patients with ectopic heartbeats >5% of all beats who were unsuitable for HRV analysis (2 patients were excluded).

This study was approved by the Clinical Medical Research Ethics Committee of The First Affiliated Hospital of Bengbu Medical College (Bengbu, Anhui, China) (registration number: 2021KY010). The experiments were performed in strict accordance with the ethical standards laid down in the 1964 Declaration of Helsinki and its later amendments. All patients were informed of the details of the purpose, procedures, risks and potential adverse effects of the experiment and signed an informed consent form.

### Data collection

ECG data collection was performed in a quiet temperature-controlled room (23°C ± 1C). The subjects were prohibited from consuming caffeine, alcohol or other autonomic nervous system stimulants within 24 h before the test. A single-lead miniature ECG recorder (version 2.8.0, Healink-R211B, Healink Ltd., Bengbu, China) with a V6 lead was used to collect a 5 min supine resting ECG before and the day after chemotherapy. The signal bandwidth of the equipment was set to 0.67–40 Hz, and the sampling rate was 400 Hz. The patients were asked to keep quiet and breathe smoothly during the period of ECG collection.

### HRV analysis

The Pan-Tompkins algorithm was used to extract the time series of the RRI. The artifacts caused by interference and ectopic heartbeat were corrected by a time-varying threshold algorithm, and then HRV time and frequency domain analysis was carried out.

The time-domain indices included the standard deviation of all normal-to-normal intervals (SDNN) and the root mean square of successive differences (RMSSD).

The frequency-domain indices included low-frequency power (LF, 0.04–0.15 Hz), high-frequency power (HF, 0.15–0.4 Hz), and the ratio of LF to HF (LF/HF).

The above analysis was performed with Kubios HRV Premium software (version 3.1.0, https://www.kubios.com, Kubios Oy, Kuopio, Finland).

### Statistical analysis

The normality of all data was checked by the Shapiro-Wilk test, and the normally distributed data are expressed as the mean ± standard deviation. The nonnormally distributed data are expressed as the median (first quartile, third quartile). Independent sample *t* tests and chi-square tests were used to assess the differences in basic clinical information between the two groups; Mann‒Whitney *U* tests were used to compare the group differences in each HRV index before chemotherapy between the two groups; and paired samples *t* tests and Wilcoxon signed rank tests were used to examine the differences in HRV indices before and after chemotherapy between the two groups. *Cohen’s d* value was used to characterize the effect size of the difference in each HRV index before and after chemotherapy, with *d* = 0.2 considered a small effect, *d* = 0.5 considered a moderate effect, and *d* = 0.8 considered a large effect (Cohen, 1992). SPSS Statistics 26.0 (IBM Corp., Chicago, Illinois, United States of America) software was used for the above statistical analysis, and *p* < 0.05 (two-tailed) was considered statistically significant.

## Results

A total of 50 patients met the inclusion criteria and were divided into two groups based on chemotherapy regimen: 19 patients in the DC group (docetaxel combined with carboplatin) and 31 patients in the TC group (paclitaxel combined with carboplatin). Table 1 summarizes the basic clinical data of the two groups, and Table 2 shows the baseline HRV of the two groups. There were no significant differences in age, BMI, mean heart rate, diabetes, hypertension, histological type or baseline HRV between the two groups (*p* > 0.05), and there was a significant difference in whether the patients were receiving chemotherapy for the first time (*p* = 0.019).

**TABLE 1:** Clinical and demographic data.

Characteristics	DC (N = 19)	TC (N = 31)	*P*
**Age** (years)	52.7 ± 10.6	55.5 ± 9.4	0.339
**BMI** (kg/m^2^)	22.9 ± 2.8	24.5 ± 3.6	0.113
**Mean HR** (bpm)	84 ± 12	89 ± 10	0.127
**Diabetes** [N (%)]			0.983
yes	1 (5.3)	3 (9.7)	
no	18 (94.7)	28 (90.3)	
**Hypertension** [N (%)]			0.160
yes	2 (10.5)	10 (32.3)	
no	17 (89.5)	21 (67.7)	
**Histological type** [N (%)]			0.560
squamous carcinoma	16 (84.2)	29 (93.5)	
adenocarcinoma	3 (15.8)	2 (6.5)	
**First chemotherapy** [N (%)]			**0.019**
yes	17 (89.5)	18 (58.1)	
no	2 (10.5)	13 (41.9)	

Abbreviations: N, number of individuals; BMI, body mass index; Mean HR, mean heart rate; bpm, beats per minute.

The bold value means that the difference is statistically significant.

**TABLE 2:** HRV parameters of the patients before chemotherapy.

HRV	DC (N = 19)	TC (N = 31)	*P*
**SDNN**	11.1 (8.5, 15.3)	9.4 (7.1, 12.5)	0.165
**RMSSD**	9.7 (7.6, 16.4)	8.2 (5.9, 14.5)	0.280
**LF**	64 (22, 116)	29 (16, 74)	0.093
**HF**	42 (22, 126)	36 (17, 87)	0.358
**LF/HF**	1.150 (0.400, 2.545)	0.890 (0.366, 1.595)	0.697

Abbreviations: N, number of individuals.

**FIGURE 1:**
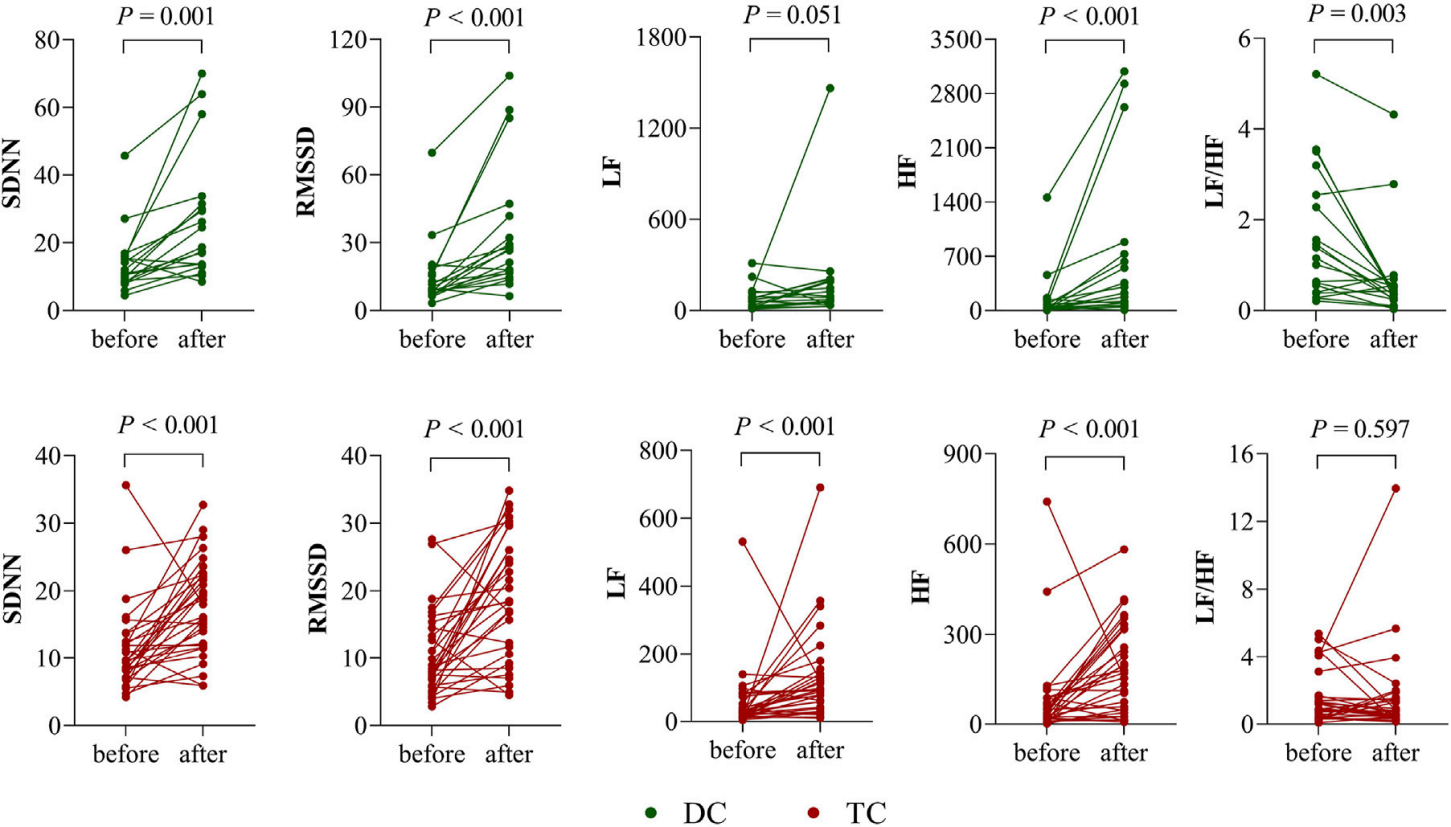
Differences in HRV before and after chemotherapy in the DC and TC groups.

Figure 1 shows the comparison of HRV parameters before and after chemotherapy in the DC and TC groups. In the DC group, SDNN, RMSSD, and HF were significantly higher after chemotherapy than before (*p* < 0.05); LF/HF was significantly lower than before (*p* < 0.05); and LF was not significantly different before and after chemotherapy (*p* > 0.05). In the TC group, SDNN, RMSSD, LF, and HF were significantly higher after chemotherapy than before (*p* < 0.05); LF/HF was not significantly different before and after chemotherapy (*p* > 0.05).

**FIGURE 2:**
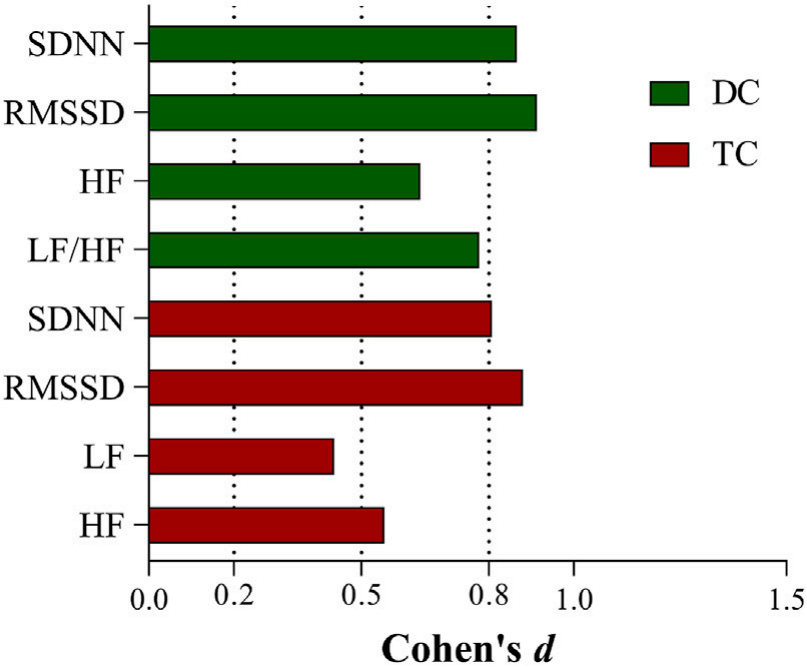
Effect sizes of HRV indicators with differences before and after chemotherapy in the DC and TC groups.

The effect sizes of the differences in each HRV parameter before and after chemotherapy in the two groups are shown in Figure 2.

## Discussion

This is a study to evaluate the effects of combined taxane and carboplatin chemotherapy on early cardiac ANS function in patients with CC. In this study, the traditional time-domain (SDNN, RMSSD) and frequency-domain (LF, HF, LF/HF) indices were used to analyze the HRV of 50 CC patients before and after chemotherapy. The results showed that significant increases in SDNN, RMSSD and HF were observed in both groups. In addition, LF increased significantly in the TC group and LF/HF decreased significantly in the DC group. The alteration of HRV predicts changes in cardiac ANS function, and the results of this study suggest that combined taxane and carboplatin chemotherapy may affect the early ANS status in CC patients.

The mechanism of the effect of taxane on cardiac function in patients has not been clarified. Because it belongs to the anti-microtubule class of drugs, it acts as an anticancer agent by promoting polymerization of tubulin, forming stable microtubules, and inhibiting cell division. However, it can damage the cytoskeleton due to its anticancer properties and impair the basic functions of cardiac endothelial cells, which in turn leads to myocardial injury (Morbidelli et al., 2016; Rosa et al., 2016). Several studies have shown that the occurrence of multiple cardiac disturbance events (including arrhythmias, bradycardia, and different degrees of atrioventricular block) can be observed during the administration of taxane (Rowinsky et al., 1991; Markman and Nazarian, 2016). The altered HRV observed in our study may be an early sign of cardiac disturbance events. There are few reports on the cardiotoxicity of carboplatin, and its major toxic side effect manifests as myelosuppression (Oun et al., 2018). However, it cannot be determined whether some of the HRV alterations observed in this study originated from the combination of carboplatin.

RMSSD is highly correlated with HF, both representing vagal activity (Picard et al., 2021). The significant increase in RMSSD and HF indicates enhanced cardiac vagal activity, whereas vagal activity stimulation shortens the atrial effective refractory period, increases spatial electrophysiological heterogeneity, and promotes early after-depolarization at the end of action potential phase 3, which may be a trigger for arrhythmias (Shen and Zipes, 2014). SDNN is generally thought to reflect the overall activity of sympathetic and vagal nerves, but the main cause of its variability in short-time recordings comes from respiratory sinus arrhythmias (RSA) (Shaffer et al., 2014; Shaffer and Ginsberg, 2017). RSA is a physiological phenomenon resulting from the regulation of the cardiac system by the vagus nerve and could be used as an indicator of vagal activity (Porges, 2007; Lubocka and Sabiniewicz, 2021). It has been found that decreased RSA is associated with reduced cardiac vagal activity in patients with paroxysmal atrial fibrillation after undergoing pulmonary vein isolation (Jungen et al., 2019). Increasing the biofeedback training of RSA can effectively enhance vagal regulation (Munafò et al., 2016). Moreover, various studies have noted that RSA is highly correlated with SDNN (Smith et al., 2013; Vieira et al., 2016). Consequently, in our study, the significant increase in SDNN after chemotherapy was most likely attributable to an increase in vagal activity rather than an alteration in overall ANS activity.

Compared to time-domain indices, the study of HRV frequency-domain indices contains a great deal of uncertainty. The physiological significance of LF has been controversial. Initially, LF was considered to reflect sympathetic activity (Pagani et al., 1997). Later, it was reported that LF represents the comodulation of the sympathetic-vagal system (Vanderlei et al., 2009). In recent years, other researchers have found that LF primarily reflects baroreflex activity (Goldstein et al., 2011; Rahman et al., 2011). This challenges the notion that LF/HF represents sympathetic-vagal balance (Billman, 2013). LF and LF/HF may not accurately reflect the state of the ANS. We observed a significant increase in LF in the TC group and a significant decrease in LF/HF in the DC group, which probably relates to the complex interaction between sympathetic and parasympathetic nerves, as well as mechanical effects caused by respiration (Billman, 2011). Additionally, in contrast to the DC group, the TC group required Cremophor EL as a solvent for the formulation of paclitaxel; evidence suggests that this solvent induces histamine release and thus causes cardiovascular stimulation (Rowinsky et al., 1991; Madeddu et al., 2016, Al-Mahayri et al., 2021), which may also be a possible reason for the elevated LF we observed in the TC group. It is worth mentioning that in our research, the effect sizes of the time-domain indicators (SDNN, RMSSD) before and after chemotherapy were greater than 0.8 in both groups. We consider the time domain is more sensitive than the frequency domain in reflecting the physiological state of the body.

In previous studies examining the effects of chemotherapeutic agents on the HRV of patients, some researchers have found no significant change in HRV in patients before and after chemotherapy. For example, Ekholm et al. included 24 BC patients previously pretreated with anthracyclines and evaluated their HRV by 24-h ambulatory ECG before and after three to four courses of docetaxel treatment. The results showed that the HRV time-domain (SDNN, NN50, RMSSD) and frequency-domain (LF, HF, VLF, LF/HF) parameters of the patients after chemotherapy were not significantly altered (Ekholm et al., 2002). In contrast to their findings, the patients in this study had significantly higher SDNN, RMSSD, and HF after chemotherapy. We suppose that, owing to the different chemotherapeutic agents from the previous studies and the heterogeneity of the study population, there will be some differences regarding the effects of HRV. Additionally, most of the present studies have used 24-h ambulatory ECGs to observe HRV in patients after several courses of treatment, and the series of changes observed may contribute to long-term cumulative drug effects. In contrast, the present study used short-term ECGs for short-term observation, and we collected 5-min ECGs before and the day after chemotherapy to analyze the HRV of patients. We considered the relatively subtle effect of chemotherapy drugs on the cardiac ANS in the short term; that is, chemotherapy drug use stimulates the patient’s cardiac vagus nerve in the short term, resulting in elevated SDNN, RMSSD and HF.

### Limitations

This explorative study included a relatively small number of patients, and there was heterogeneity in the dosage of chemotherapeutic drugs. Furthermore, our study merely observed short-term changes in HRV in patients and did not indicate whether these changes are permanent. Therefore, the results of this study need to be confirmed in a prospective study with a larger sample size, homogeneous drug doses and a long-term follow-up period.

## Conclusion

Our findings demonstrate that combined taxane and carboplatin chemotherapy can increase HRV in the short term for CC patients. HRV may be a sensitive tool for the early detection of cardiac ANS perturbations caused by chemotherapy with taxane combined with carboplatin. Early changes in cardiac function can be monitored clinically based on HRV alterations to prevent cardiotoxicity and myocardial injury in patients.

## Data Availability

The raw data supporting the conclusion of this article will be made available by the authors, without undue reservation.
